# Efficient Eradication of Mature *Pseudomonas aeruginosa* Biofilm via Controlled Delivery of Nitric Oxide Combined with Antimicrobial Peptide and Antibiotics

**DOI:** 10.3389/fmicb.2016.01260

**Published:** 2016-08-17

**Authors:** Hang Ren, Jianfeng Wu, Alessandro Colletta, Mark E. Meyerhoff, Chuanwu Xi

**Affiliations:** ^1^Department of Chemistry, University of Michigan, Ann Arbor, MIUSA; ^2^Department of Environmental Health Sciences, School of Public Health, University of Michigan, Ann Arbor, MIUSA

**Keywords:** nitric oxide, antimicrobial agents, combination, biofilms, *Pseudomonas aeruginosa*, antimicrobial peptide, antibiotics

## Abstract

Fast eradication of mature biofilms is the ‘holy grail’ in the clinical management of device-related infections. Endogenous nitric oxide (NO) produced by macrophages plays an important role in host defense against intracellular pathogens, and NO is a promising agent in preventing biofilms formation *in vitro*. However, the rate of delivery of NO by various NO donors (e.g., diazeniumdiolates, *S*-nitrosothiols, etc.) is difficult to control, which hinders fundamental studies aimed at understanding the role of NO in biofilm control. In this study, by using a novel precisely controlled electrochemical NO releasing catheter device, we examine the effect of physiological levels of NO on eradicating mature *Pseudomonas aeruginosa* biofilm (7 days), as well as the potential application of the combination of NO with antimicrobial agents. It is shown that physiological levels of NO exhibit mixed effects of killing bacteria and dispersing ambient biofilm. The overall biofilm-eradicating effect of NO is quite efficient in a dose-dependent manner over a 3 h period of NO treatment. Moreover, NO also greatly enhances the efficacy of antimicrobial agents, including human beta-defensin 2 (BD-2) and several antibiotics, in eradicating biofilm and its detached cells, which otherwise exhibited high recalcitrance to these antimicrobial agents. The electrochemical NO release technology offers a powerful tool in evaluating the role of NO in biofilm control as well as a promising approach when combined with antimicrobial agents to treat biofilm-associated infections in hospital settings, especially infections resulting from intravascular catheters.

## Introduction

Implanted medical devices are frequently used in hospitals for therapeutic and diagnostic purposes. However, the use of many of these devices, especially indwelling ones such as intravascular catheters, are often associated with increased risk of infection (Donlan, 2001). Antibiotic lock solutions and systemic usage of antibiotics is a common clinical practice (Del Pozo et al., 2012), but biofilm can still form on the devices, and the rate of treatment failure can reach 50% depending on the factors of the pathogens and the host (Baldassarri et al., 2006). Once the biofilm matures (>96 h), the bacteria in the biofilm show severe recalcitrance to antibiotic treatment as most antibiotics target planktonic bacteria rather than those in the biofilm (Wolcott et al., 2010). Many studies have focused on the prevention of biofilm formation by obstructing the initial attachment, either by coating antibiotic on the surface or by modifying the physio-chemical properties of the surface (Hasan et al., 2013). However, these strategies fail to stop the biofilm formation completely as the desired surface property can be dramatically masked or changed in the clinical setting. In addition, many prevention strategies rely on sustained treatment, which adds additional pain and medical cost to the patients. Strategies to eradicate mature biofilm on medical devices are considered the “holy grail” in controlling biofilm-related infections.

To address the problem of mature biofilms on devices, various strategies including use of biofilm-breaking enzymes (Chaignon et al., 2007), quorum sensing agents (Cirioni et al., 2006), and bacteriophages (Hughes et al., 1998) have been attempted. However, they suffer from being restricted to a spectrum of microbes on which they are effective, as well as the potential for toxicity or immune response *in vivo*. To circumvent these limitations, nitric oxide (NO) is attractive as it is endogenously produced and participates in the human host defense system. Various polymeric materials capable of NO release at 0.5–4.0 × 10^-10^ mol cm^-2^ min^-1^ (0.5–4.0 flux) have been developed over the past two decades because this range is considered physiologically relevant and safe (Vaughn et al., 1998; Frost et al., 2005; Carpenter and Schoenfisch, 2012). However, the effectiveness of NO in this frequently targeted range on mature biofilm is not entirely clear. In most cases, NO starts to release as soon as the materials are introduced to the bacteria, demonstrating the prevention of biofilm rather than the eradication of mature biofilm.

To study the effect of NO on mature biofilm, the direct use of NO donors on mature biofilm has received some attention (Barraud et al., 2006, 2009a,b; de la Fuente-Nunez et al., 2013; Alexander et al., 2015); however, the observed effect of NO is often influenced by the high concentration of NO donors in the media (up 1000× greater than NO). Hence, the organic portion of the donor and its degradation product can contribute to the observed effect (Feelisch, 1998; Frost et al., 2005). In addition, the release of NO from donors in the medium is less controlled and is strongly influenced by the medium composition, including pH, ionic strength and even trace amount of metal ions (Wang et al., 2002; Frost et al., 2005; Harding and Reynolds, 2014). Therefore, it is desirable to elucidate the quantitative effect of NO on mature bacterial biofilms via a predictable and controllable NO release system, without exposing the bacteria to NO prior to biofilm formation, as well as to any organic NO donor molecule or associated product species after NO release.

In addition, the fate of NO treated bacteria and the interaction of NO release with host defense and routinely applied antibiotics are also critical for the application of any NO release technique in future medical devices. Previous studies on the utility of NO on the efficacy of antibiotics have yielded mixed results (Barraud et al., 2009b; Gusarov et al., 2009; Privett et al., 2010; McCollister et al., 2011; Zemke et al., 2015). Therefore, any potential synergistic effects between NO and endogenous and exogenous antimicrobial agents needs careful investigation.

A recently developed electrochemical NO release catheter (Ren et al., 2014) provides an ideal tool for these studies. In this system, NO can be released on demand from the surface of a catheter or other device with highly tunable and predictable fluxes, free from the interference of any organic NO donor structures that could also affect the bacteria. Using this NO release model, the prevention of biofilm formation has been demonstrated (Ren et al., 2014, 2015). In the current study, we further establish the dosage effects of physiological levels of NO release on eradicating mature *Pseudomonas aeruginosa* biofilm using the electrochemical NO release catheter. The potential application of the combination of NO release with antibacterial agents is also evaluated. The goal is to eradicate detached cells from biofilms to prevent dispersal of bacterial cells to other sites causing secondary infections. Our approach is that by disrupting bacterial biofilms into dispersed bacterial cells via NO treatment, these bacteria can be further eradicated by the human immune system or conventional antibiotics, which would offer a new therapeutic approach for disease treatment (Bordi and de Bentzmann, 2011).

## Materials and Methods

### Catheter Fabrication and NO Release Profile Measurements

**FIGURE 1:**
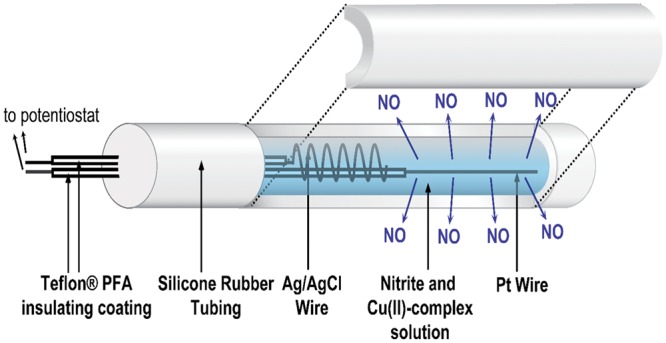
**Schematic of the electrochemical NO releasing catheter employed in this study, with a cutaway view showing the inner electrodes of the catheter that are capable of creating NO from inorganic nitrite via electrochemical reduction reaction mediated by a Cu(II)-ligand complex**.

The catheter fabrication procedures used were similar to those reported previously (Ren et al., 2014, 2015). A single lumen silicone tube (o.d. 1.96 mm, i.d. 1.47 mm) was cut into 6 cm lengths, and each piece was sealed at one end with silicone rubber adhesive (3140 RTV, Dow-Corning, Midland, MI, USA). The lumen was filled with a solution containing 4 mM copper(II)-tri(2-pyridylmethyl)amine, 0.4 M NaNO_2_, 0.2 M NaCl, and 0.5 M HEPES buffer (pH 7.2). A Teflon-coated Pt wire (3 cm exposed) and a Ag/AgCl wire (5 cm exposed) were inserted into the lumen as the working and reference/counter electrodes, respectively. The opening of the lumen at the proximal end was then sealed (around the wires) with silicone rubber adhesive and left to cure in water overnight (see **Figure 1**). The NO release profile of the catheters was tested by applying different voltages, and the NO flux from the surface of the catheters was quantitated using a NO chemiluminescence analyzer (Sievers 280i, GE Analytics, Boulder, CO, USA), as reported previously (Zheng et al., 2015).

### Bacterial Strain and Biofilm Growth

*Pseudomonas aeruginosa* PAO1 wild-type strain was obtained from University of Washington (UW Genome Sciences, Seattle, WA, USA; Winsor et al., 2011). The bacterial strain was maintained on a Luria Bertani (LB) agar plate and grown in LB broth. Biofilms were developed on the outer surface of the catheter tubing in a CDC bioreactor (BioSurface Technologies, Bozeman, MT, USA) supplemented with 10% strength of LB broth. Briefly, the electrochemical NO release catheters were mounted on the holders within the CDC bioreactor. Four mL of overnight grown *P. aeruginosa* PAO1 culture were inoculated into the CDC bioreactor at final concentration about 10^6^ CFU/mL, and the CDC bioreactor was left static for 1 h before introducing fresh 10% LB media at 100 mL/h via a peristaltic pump and starting the magnetic stirrer to generate shear force (300 rpm, ~0.08 N m^-2^; Goeres et al., 2005). The biofilms were allowed to develop on the surface of the catheters in the bioreactor for 7 days (d) at 37°C, and the catheter pieces were then taken out aseptically from the reactor and gently rinsed in sterile PBS to remove any loosely attached bacteria. The catheters were then subjected to further studies.

### Dosage Effect of NO on 7-day Biofilms Disruption

**FIGURE 2:**
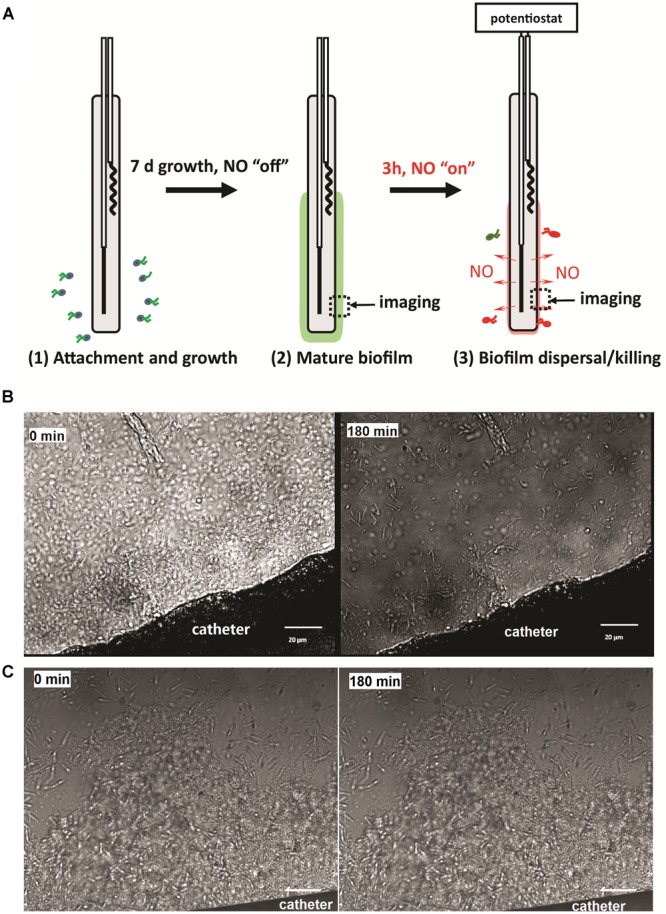
**(A)** Illustration of the experimental procedure used in this study; **(B)** representative images of NO release catheter surface and its biofilm at 0 and 3 h. NO release from the surfaces of the catheters at a flux of 1.5 × 10^-10^ mol cm^-2^ min^-1^; **(C)** representative images of biofilm on control catheters at 0 and 3 h. Green dash line indicates the edge of biofilm. Black arrow shows the location of biofilm.

The catheters with 7-day biofilms were transferred into 5 mL of PBS in a 15-mL centrifuge tube. The wires of the catheters were connected to a multi-channel potentiostat (1000C, CH Instrument, Austin, TX, USA), with the platinum wire connected to the working electrode lead, and the silver wire to the reference and counter leads. The NO release was then turned “on” for 3 h by applying the voltages required to achieve the flux desired at the outer surface of the catheters (e.g., -0.22 V for 0.3 flux, -0.23 V for 0.5 flux, -0.275 V for 1.5 flux, and -0.325 V for 3.0 flux; Ren et al., 2014). The solution remained static during the dispersal experiment (**Figure 2A**). After 3 h of NO release, the viable bacterial cells remaining on the catheter surfaces were quantified by plate counts. Briefly, the catheters were taken out of the PBS, and the inner filling solutions of the catheters were carefully removed using a syringe from the proximal end of the catheters. A 3 cm piece of the catheter was cut off and put into a 2 mL fresh PBS and treated with a homogenizer (Omni International, Kennesaw, GA, USA) at the highest speed for 1 min to remove and homogenize the biofilm from the catheter surface into PBS. The homogenate was 10-fold serially diluted with PBS, and 50 μL of each dilution was plated onto LB agar plates, that were incubated overnight at 37°C for the colony-forming unit (CFU) counting.

The bacteria released to the buffer during the 3 h NO release experiment were also quantified similarly as described above. The buffer was homogenized before serial dilutions and plate counts. The catheter samples at each flux level were tested in triplicate, and the Student’s *t*-test was used for statistical analysis. The total viable bacterial number of the biofilm was calculated by adding the number of bacteria on the catheter and the number of bacteria in the buffer.

### Susceptibility of *P. aeruginosa* Biofilm to Antimicrobial Agents Combined with NO Release

The procedures were similar to those of the dosage study described above. The 7 days biofilm attached catheters were placed in PBS containing 20 μg/mL of beta-defensin 2 (BD-2), or 100 μg/mL of antibiotic. The antibiotics examined included colistin, gentamicin, chloramphenicol, ciprofloxacin and tetracycline (*n* = 3 catheters for each condition). NO was turned “on” at 1.5 flux immediately after the catheters were introduced to the buffer containing the antimicrobial agents. Catheters without turning “on” the NO release were used as controls. After a 3 h incubation, the bacteria remaining on the catheter surfaces as well as those dispersed in the buffer were quantified by plate counting as described above.

### Susceptibility of *P. aeruginosa* Released Cells From Biofilms to Antimicrobial Agents Combined with NO Release

To further confirm the increased susceptibility of the biofilm-detached cells, another set of catheters with pre-developed biofilms were placed in PBS without gentamicin/NO for 3 h to let the biofilm bacteria shed naturally. The PBS was then homogenized as described above and treated with gentamicin at various concentrations (0–500 μg/mL) in the presence and absence of NO for 3 h. Viable bacteria in buffer were enumerated by plate counting. Experiments were performed in triplicate.

### Statistical Methods

All data were presented as the mean value with their standard deviation of replicates of different biological samples with *n* = 3. The geometric mean was used for the log transformation of the bacterial plate counting data. Student’s *t*-test was used for calculating the confidence intervals and significance. *p*-values were calculated from *t* distribution with degree of freedom of 2.

## Results

### Physiological Levels of NO Effectively Eradicated 7 days *P. aeruginosa* Biofilm

**FIGURE 3:**
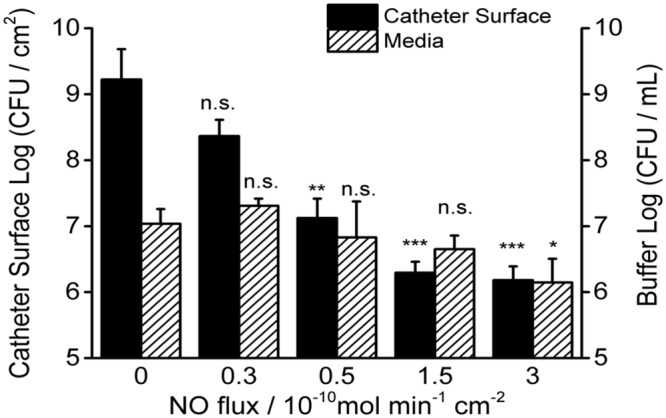
**Dosage effects of 3 h NO release on 7-day *P. aeruginosa* biofilms.** Viable bacteria were measured both on the surface of catheters and in the buffer after NO turned “on” for 3 h at room temperature. Asterisk denotes statistical significance at each flux compared with corresponding 0 flux (*n* = 3 catheters for each flux, n.s.: no significance, *p < 0.05; **p < 0.01, ***p < 0.001).

To study the effect of physiological levels of NO on mature biofilm, *P. aeruginosa* biofilm was allowed to develop on the surface of the catheters for 7 days before NO was turned “on” (NO release profile demonstrated in Supplementary Figure S1). As shown in **Figure 2B**, 3 h of NO release at 1.5 flux induced removal of mature biofilms from the catheter surface. While the biofilm remained intact on the control catheter’s surface (**Figure 2C**). To further evaluate the overall eradicating effect, each of the catheters with pre-developed *P. aeruginosa* biofilms was transferred into separate PBS solutions. Different levels of NO were then turned “on” for 3 h, and the viable bacteria on the catheter surfaces (in biofilms) and those detached/dispersed into the buffer were quantified by the plate counting methods. From **Figure 3**, it can be seen that there were significant levels of viable bacteria within the 7-days biofilm and it naturally dispersed cells (about 1% of total biofilm cells) from biofilm matrix to the nearby buffer solution. When NO was turned on, at only 0.3 flux, the NO release already showed a significant biofilm eradicating effect by reducing 86% of the viable biofilm on the surface.

The biofilm eradicating effect was positively correlated with NO flux and reached a 3-log reduction when the NO flux was >1.5 (see **Figure 3** and **Table 1**). As for the cells in the nearby buffer, the NO showed the same trend as observed for its effect on the surface biofilm, except when NO was at the flux of 0.3, it increased the number of dispersed cells in buffer than biofilm naturally dispersed (87%) and the difference was close to being significant (*p* = 0.06). This result indicates that the effect of NO on biofilm is a mixed process, with both killing and dispersal effects. The total viable cells (the sum of the viable bacteria on the catheter surface and those dispersed into the buffer) decreased with an increase in NO release flux. More than a 1-log reduction of the total viable cells was observed in 3 h when the NO was above 0.5 flux, and a 2-log reduction was found when NO was at 3.0 flux, indicating the significant killing effect of physiological levels of NO on biofilms. However, only a modest killing effect (reduction within 1 log) was observed when NO was turned “on” to treat the bacteria in the buffer. This is likely due to the short half-life of NO as it diffuses out into the surrounding solution, owing to its reaction with oxygen.

**Table 1:** Effect of NO flux on dispersing and killing of 7-day *Pseudomonas aeruginosa* biofilm.^†^

NO flux/10^-10^ mol cm^-2^ min^-1^	0	0.3	0.5	1.5	3.0
Attached viable cells	96.5%	13.4%	0.8%	0.1%	0.1%
Detached viable cells	3.5%	6.6%	2.2%	1.4%	0.5%
Total viable cells	100.0%	20.0%	2.9%	1.6%	0.5%

*^†^The data is normalized to total viable cell from control (0 flux of NO). Total viable cells are calculated by adding the number of viable cells on the catheter (CFU/cm^2^ multiplied by the surface area of the catheter) and those in the buffer (CFU/mL multiplied by the volume of buffer).*

### NO Increases Susceptibility of *P. aeruginosa* Biofilm to Endogenous and Exogenous Antimicrobial Agents

**FIGURE 4:**
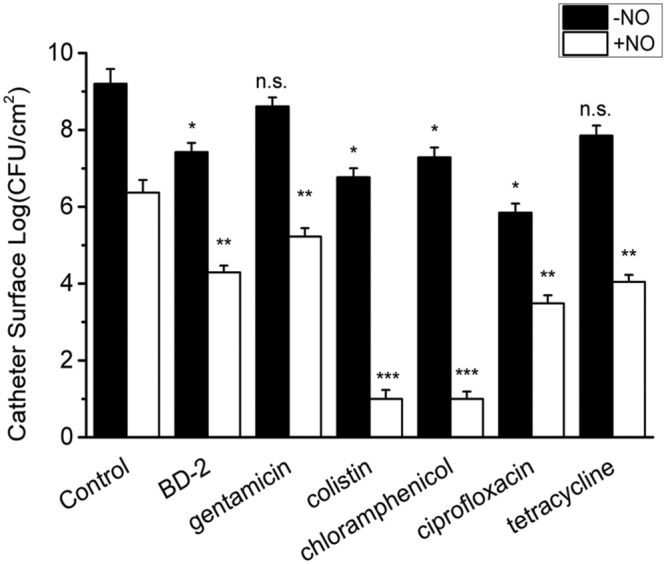
**Efficacy of 3 h treatment of BD-2 and various antibiotics against 7-day *P. aeruginosa* biofilm in the absence and presence of NO release.** The concentration of BD-2 is 20 μg/mL, and concentration for all other antibiotics is 100 μg/mL. NO release is 1.5 flux. Asterisk denotes statistical significance compared with the control samples (*n* = 3 catheters for treatment, n.s., no significance, *p < 0.05; **p < 0.01, ***p < 0.001).

Since NO at physiological levels showed a significant killing effect on the biofilm cells, the interaction of NO with other antimicrobial agents was also evaluated. We first studied the combination of NO and BD-2, as BD-2 is an important endogenous antimicrobial peptide in human host defense. In the absence of NO, the biofilm showed recalcitrance (<1 log reduction) to BD-2 (see **Figure 4**). Interestingly, NO showed a strong enhancing effect with BD-2 (at 20 μg/mL) in eradicating pre-developed *P. aeruginosa* biofilm. The combination of NO release at 1.5 flux and BD-2 can induce 4-log reduction in 3 h.

Similarly, a significant enhancing effect of NO release on antibiotic activity was observed for gentamicin, colistin, chloramphenicol, ciprofloxacin and tetracycline, when each was present at 10 μg/mL. In the absence of NO release, *P. aeruginosa* biofilm showed significant recalcitrance to all the antibiotics at 100 μg/mL. When NO was turned “on” at 1.5 flux, the biofilm cells became much more susceptible to antibiotics, resulting >3 log reduction of viable biofilms counts in 3 h for all the antibiotics (see **Figure 4**). For colistin and chloramphenicol, biofilm was completely eradicated in 3 h when combined with NO release (viable cells are below the detection limit for plate counting method, <10 CFU/mL). The combination of each antibiotic with NO showed a significantly greater reduction in viable biofilm cells than NO or gentamicin acting alone or even their sum, indicating a significant enhancing effect of NO on the efficacy of antibiotics against biofilms (Supplementary Table S1).

### NO Increases Susceptibility of *P. aeruginosa* Biofilm Released Cells to Antimicrobials

**FIGURE 5:**
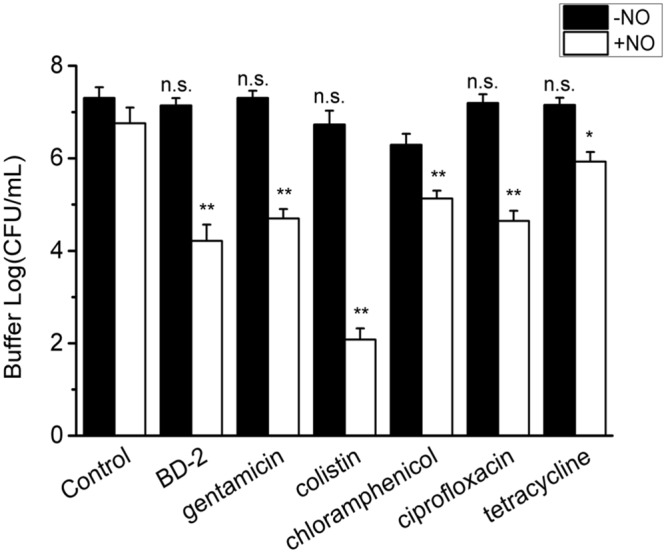
**Efficacy of 3 h treatment of BD-2 and antibiotics against 7-day *P. aeruginosa* biofilm released cells in PBS in the absence and presence of NO release.** The concentration of BD-2 is 20 μg/mL, and concentration for all other antibiotics is 100 μg/mL. NO flux is 1.5. Asterisk denotes statistical significance compared with the control samples (*n* = 3 catheters for treatment, n.s., no significance, *p < 0.05; **p < 0.01).

Surprisingly, released cells from the biofilm in the buffer also show high recalcitrance to antibiotics in the absence of NO (<1 log reduction). However, the enhanced efficacy from the combination of NO and BD-2 and other antibiotics against these cells in the buffer is still very evident. As shown in **Figure 5**, a total of 4 log reduction was observed for the combination of BD-2 and NO release in 3 h.

The efficacy of the other antibiotics is also generally improved by NO release. Colistin combined with NO yielded a 5 log reduction of the biofilm released cells in the buffer (see **Figure 5**). For gentamicin, chloramphenicol and ciprofloxacin, the reduction was >2 log in the presence of NO. The enhancing effect from the combination of NO and antibiotic was again confirmed by the significant difference (>1 log) between the log reduction of viable cells from combined treatment and the sum of that from individual treatment with *p* < 0.01 for BD-2 and all the antibiotic tested except for tetracycline. Similarly, the antibiotics act in a dose-response manner in the presence of NO.

## Discussion

Numerous of strategies have been developed for controlling biofilm on medical devices. Ideally, preventing biofilm formation would be a more logical option than treating it (Simões et al., 2010). However, in the clinical setting, biofilm disruption or removal, though notoriously difficult, is as important as biofilm prevention since patients cannot take medication in perpetuity to avoid biofilm formation. In this study, we first established the dosage effect of physiological levels of NO on pre-formed biofilms. Further, the effectiveness of NO with an endogenous antimicrobial peptide and other classical antibiotics in killing biofilms was evaluated. Our results show that NO release at physiological fluxes is effective in reducing pre-formed biofilms; moreover, the used of NO in combination with antimicrobial agents, including antimicrobial peptide and antibiotics, yields an enhancing killing effect. This confirms our hypothesis that physiological levels of NO have the capacity of killing/dispersing pre-developed mature biofilms, and the NO-dispersed cells are more susceptible to antimicrobial peptides or antibiotic. This result is encouraging as it indicates that the function of NO release is not limited to dispersal or killing of biofilms: NO release can render the bacteria more susceptible to the host defense system and to conventional antibiotic treatment.

One finding from this study is that biofilm cells dispersed from the biofilm matrix into the ambient environment have different properties from their planktonic form in terms of antibiotic susceptibility. Those dispersed cells show stronger recalcitrance to antimicrobial agents than planktonic cells. This result suggests that for the biofilm-associated infections in hospitals, the challenge might not be just the biofilm themselves. Although, we currently do not know whether this property is inheritable or only temporarily displayed, these highly drug-resistant cells dispersed from the biofilms deserve additional attention, as they may circulate and grow inside the body, causing secondary or systemic infections while being far less sensitive to traditional antibiotic treatment. In this study, interestingly, we found that if these detached cells were dispersed by NO from the biofilm matrix or exposed to NO, their drug-resistance is greatly attenuated (**Figure 5**). These data strongly support our hypothesis that by using NO to disperse biofilm cells and then using a conventional antimicrobial agent method, these floating cells can be killed more effectively. Moreover, synergy was observed between NO and antibiotics against both biofilm and planktonic cells, suggesting that NO release from an implanted medical device at physiological levels is a safe and promising means of controlling the challenging problem of biofilms on such devices. Indeed, the local release of physiological levels of NO on indwelling medical devices could potentially decrease the length of treatment as well as the dosage of antibiotics required for successful treatment of device-related infections, offering a potential solution to the clinical crisis of biofilm-associated infections.

**FIGURE 6:**
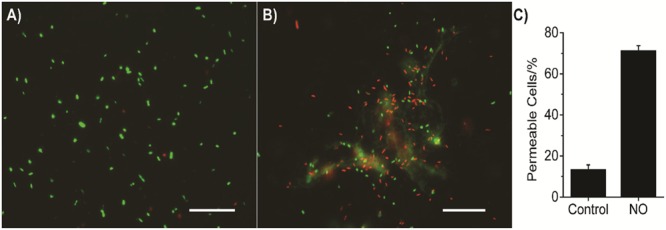
**Representative images of *P. aeruginosa* biofilm released cells staining with Live/Dead BacLight^TM^ Bacterial Viability kit (L7012, ThermoFisher Scientific, Waltham, MA, USA).** Biofilm released cells were stained with two fluorescent dyes, SYTO-9 and propidium iodide (PI), according to its instructions. Bacterial cells with intact membrane stain with SYTO-9 showing green fluorescent light, while cells with impaired membrane stain with PI showing red fluorescent light. **(A)** Bacteria with no NO release treatment; **(B)** Bacteria with 3 h NO release treatment; **(C)** Summary of the percentage of permeable cells (red fluorescence), error bars indicate the standard deviation of measurements from different images. The scale bars in **(A,B)** are 20 μm.

The combination of antibiotic and NO against bacteria has been tested and showed a promising result (Reffuveille et al., 2015). But the mechanism of synergy between NO and antibiotics on killing drug-sensitive/resistant bacteria in such a short time period is not entirely clear yet. The combined effects of NO with colistin, BD-2, chloramphenicol, or gentamicin are stronger than that of NO with other antibiotics. Colistin and BD-2 have similar mechanisms of action; they both interact with the bacterial membrane. Resistance to chloramphenicol and gentamicin is greatly impacted by bacterial membrane permeability (Mingeot-Leclercq et al., 1999; Delcour, 2009). Therefore, it is likely that in addition to dispersing the biofilms, NO changes the permeability of the bacterial membranes, resulting in more uptake of the antimicrobial agents. Our data suggest a 58% increase in cells with damaged membranes when these biofilm-released bacteria are subjected to NO release for 3 h (see **Figure 6**).

The effect observed from the electrochemical NO release system employed here should be differentiated from the bioelectric effect. A bioelectric effect requires bacteria experiencing an electric field of 1–20 V/cm when the bacteria directly contact the electrodes (Costerton et al., 1994; Del Pozo et al., 2008). In our system, in contrast, the biofilms are insulated by the relatively thick silicone rubber wall of the catheter and the electric field is no greater than the typical ambient electric field in most laboratories (Nair et al., 1989). The absence of significant bioelectric effect was also experimentally confirmed by similar biofilms formed on the surface (10^9^ CFU/mL) of a catheter-type electrochemical O_2_ sensor, which passes similar levels of current (several μA) but generates no NO.

It should be noted that the biofilms examined in this study were grown in the biofilm reactor *in vitro* using *P. aeruginosa* as a model. *P. aeruginosa* biofilm, a major cause of catheter associated urinary tract infections (CAUTIs) and pneumonia in hospitals, has significant clinical relevance (Jones, 2010; Sievert et al., 2013). Even though it has been reported that *P. aeruginosa* bears NO reductase that could neutralize the effect of NO (Kakishima et al., 2007), in our experiments, we found that physiological levels of NO still yielded a significant antimicrobial effect. The results warrant continuous examination of our hypothesis on different clinical pathogenic organisms, especially “ESKAPE” (*Enterococcus faecium, Staphylococcus aureus, Klebsiella pneumoniae, Acinetobacter, P. aeruginosa*, and *Enterobacter*) pathogens, which is necessary to demonstrate the wide applicability of our test system against microbes most often associated with common hospital-acquired infections (Rice, 2010). Further studies employing animal models are also warranted to demonstrate the applicability of the system in clinical settings for control of biofilm-related infections on intravascular and urinary catheters.

In summary, the precise control of the electrochemical NO release from a catheter tubing surface can provide a powerful tool to conduct fundamental studies on the true effect of NO on bacteria and their biofilms. Dosage studies revealed a significant killing effect on biofilms by NO at physiological fluxes (>0.5 × 10^-10^ mol cm^-2^ min^-1^). Such NO release also exhibits synergy with both endogenous antimicrobial peptides and various common antibiotics against biofilm and biofilm released cells. Such a combination therapy of using NO release with existing antibiotics should provide a promising new treatment approach for medical device-related infections.

## Author Contributions

HR and JW contributed equally to the work. HR, JW, MM, and CX designed the research; HR, JW, and AC performed the research; HR and JW analyzed data; HR, JW, MM, and CX wrote the manuscript.

## Conflict of Interest Statement

The authors declare that the research was conducted in the absence of any commercial or financial relationships that could be construed as a potential conflict of interest.
