# Supplementary material for: Efficient Eradication of Mature Pseudomonas aeruginosa Biofilm via Controlled Delivery of Nitric Oxide Combined with Antimicrobial Peptide and Antibiotics
Source: Front Microbiol. 2016 Aug 17;7:1260. doi: 10.3389/fmicb.2016.01260 (PMC4988120; doi:10.3389/fmicb.2016.01260)
Supplement: Supplementary file 1 [file Table_1.DOCX]

Supplementary Material

Efficient treatment of mature Pseudomonas aeruginosa biofilm via controlled delivery of nitric oxide combined with antimicrobial peptide and antibiotics

Hang Ren, Jianfeng Wu, Alessandro Colletta, Mark E. Meyerhoff, Chuanwu Xi^*^

*** Correspondence:** Chuanwu Xi: [cxi@umich.edu](mailto:cxi@umich.edu)

# Profile of NO release from electrochemical NO release catheters.

The relationship between applied voltages and NO fluxes from the catheter surface were established. As shown in Figure S1, these catheters exhibit a very steady NO release profile under a given applied voltage. Furthermore, the flux of NO from the tubing surface could be readily modulated in the range from 0 – 3.2 flux, simply by applying different voltages to the electrode wires, and the response time for this modulation is relatively fast, usually within minutes. Such precise and flexible control of the electrochemical system conveniently allows biofilms to be grown on the surface with or without exposure to NO, and the NO release can be turned “on” only at appropriate time, with a certain flux and for a given duration that are all predetermined by the study.

**
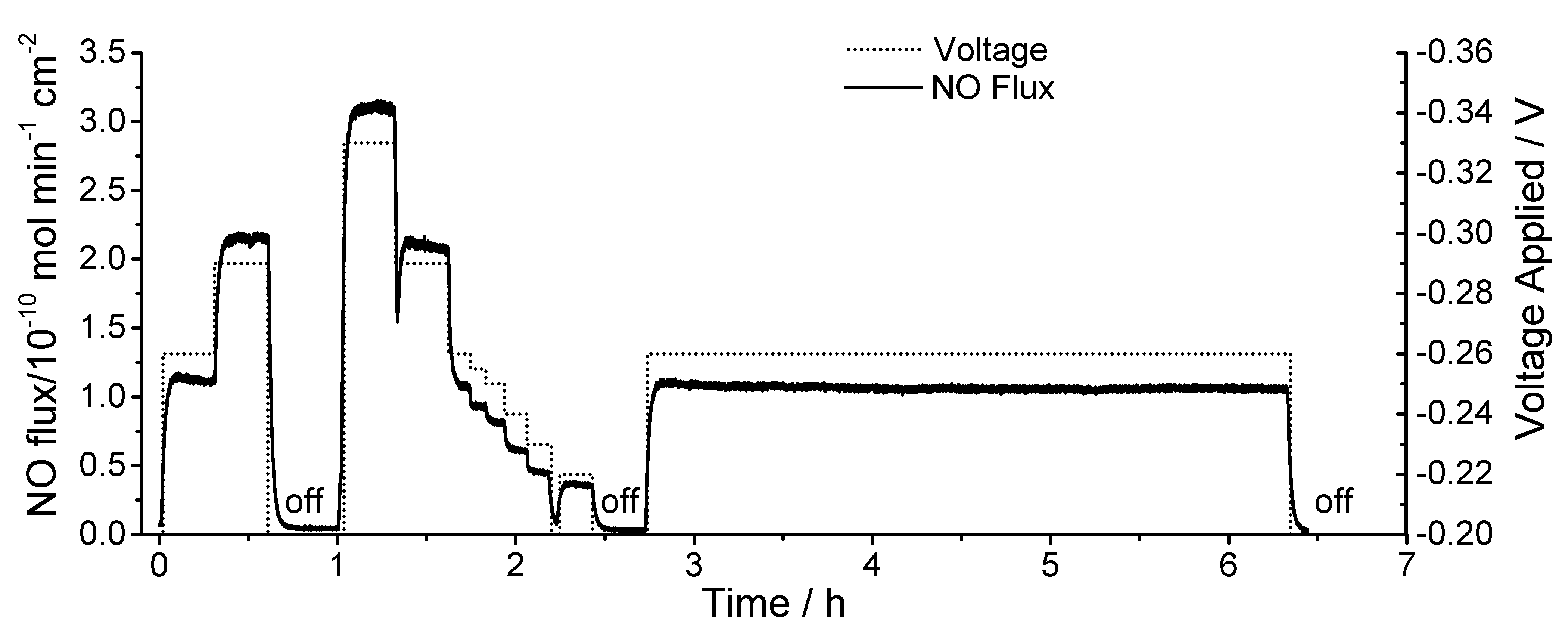
**

**Figure S1.** Modulation of surface NO fluxes from an electrochemical NO releasing catheter by different applied voltages applied to the metal wire electrodes.

Table S1. Log reduction (Δ) of viable *P. aeruginosa* biofilm from combined NO and gentamicin treatment compared with the sum of individual treatments^†^

| Gentamicin  /µg mL^-1^ | Δ for gentamicin | Sum of Δ for gentamicin and Δ for NO | Δ for combination of NO and gentamicin | Δ for synergy^‡^ | *p* value for synergy |
| --- | --- | --- | --- | --- | --- |
| 20 | 0.0 | -2.0 | -2.6 | -0.6 | 0.05 |
| 100 | -0.4 | -2.4 | -3.2 | -0.8 | 0.04 * |
| 500 | -0.8 | -2.8 | -4.9 | -2.1 | 0.01 * |

^†^ The 7 d *P. aeruginosa* biofilm was treated with different concentrations of gentamicin and/or 1.5 flux of NO release for 3 h. Δ is the log difference before and after the treatment. Initial biofilm before the treatment is ~ 10^9^ CFU/cm^2^.

^‡^ Difference between log reduction from combination treatment and the sum of that from individual treatment

* p< 0.05
